# Click Chemistry‐Based Bioconjugation of Iron Oxide Nanoparticles

**DOI:** 10.1002/smll.202407883

**Published:** 2025-02-09

**Authors:** Shno Asad, David Ahl, Yael del Carmen Suárez‐López, Máté Erdélyi, Mia Phillipson, Alexandra Teleki

**Affiliations:** ^1^ Department of Pharmacy Science for Life Laboratory Uppsala University Uppsala SE‐75123 Sweden; ^2^ Department of Medical Cell Biology Science for Life Laboratory Uppsala University Uppsala SE‐75123 Sweden; ^3^ Department of Chemistry – BMC Uppsala University Uppsala SE‐75123 Sweden

**Keywords:** active targeting, antibody, diagnostics, gastrointestinal tract, inflammatory bowel disease

## Abstract

Superparamagnetic iron oxide nanoparticles (SPIONs) exhibit unique properties for diverse biomedical applications, including drug delivery and diagnostic imaging. Actively targeted SPIONs enhance delivery to diseased sites, reducing side effects and enhancing treatment efficacy. However, the development of reproducible functionalization protocols is challenged by the erratic behavior of nanoparticles in suspensions, such as agglomeration and sedimentation. In this study, a functionalization method is developed and systematically optimized to attach the Fc‐region of antibodies onto silica‐coated SPIONs via click chemistry, ensuring controlled ligand orientation on the particle surface. The synthesis and successive modifications of silica‐coated SPIONs with organic moieties are presented resulting in the final click conjugation with antibodies targeting intercellular adhesion molecule 1 (ICAM1). This protein is upregulated on epithelial cell surfaces during gastrointestinal inflammation. Thermogravimetric analysis and infrared spectroscopy confirm successful SPION functionalization after each modification step. Cell viability assessment indicates no adverse effects of bioconjugated particles. Quantitative elemental analysis reveals significantly higher iron concentration in inflammation‐induced Caco‐2 cells exposed to ICAM1‐modified particles compared to non‐conjugated counterparts. Furthermore, laser scanning confocal microscopy of these cells suggests surface interaction and internalization of bioconjugated SPIONs, underscoring their potential for targeted imaging and therapy in inflammatory diseases.

## Graphical abstract

Inflamed intestinal cells are effectively targeted using ICAM‐1 antibody‐conjugated superparamagnetic iron oxide nanoparticles (SPIONs). Click chemistry ensures covalent, orientation‐controlled attachment of antibodies on SPIONs, significantly enhancing cellular uptake and targeting in inflamed intestinal models. This reproducible protocol highlights the potential of SPIONs for precision imaging and therapy in inflammatory diseases.

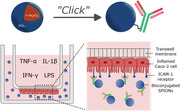

## 1 Introduction

The application of nanotechnology in medicine has yielded great developments in drug delivery and disease treatment. Clinically approved products such as Doxil, Abraxane, and Feraheme are examples of nanoparticle‐based formulations used for indications ranging from cancer to iron deficiency.^[^ ^1^ ^]^ With nanoparticle‐based mRNA vaccines against COVID‐19, the utility of nanomedical products as drug delivery vehicles has become widely adopted and well‐understood over the past years. However, there is still a clinically untapped potential for using nanoparticles, particularly metal‐based ones, for diagnosing and monitoring diseases in applications including, e.g., tissue engineering, hyperthermia cancer therapy, and magnetic resonance imaging (MRI).^[^ ^2^, ^3^ ^]^ Such applications necessitate active targeting of nanoparticles to the diseased tissue to maximize their efficacy.^[^ ^4^, ^5^, ^6^, ^7^, ^8^, ^9^, ^10^ ^]^ Advantages of functionalizing particle surfaces with coatings or ligands include improving particle stability, avoidance of protein corona formation, or enhancing therapeutic efficacy.

There are a variety of ligands that can be attached to the nanoparticle surface, including aptamers, peptides, small molecules (such as vitamins, selectin, and curcumin), carbohydrates, antibodies, and antibody fragments.^[^ ^11^ ^]^ The latter two are known for their high binding affinity, specificity, and selectivity. Antibody conjugation onto the nanoparticle surface can combine the highly specific recognition ability of antibodies with the functionality of nanoparticles (e.g., as contrast agents for MRI), making them a promising system for targeted biomedical applications.^[^ ^8^, ^9^, ^10^ ^]^ This is supported by the surge of potential disease targets identified in recent years by omics‐based technologies, paving the way for precision medicine utilizing targeted nanoparticles. One potential application area of bioconjugated nanoparticles is the treatment or diagnosis of diseases affecting the gastrointestinal (GI) tract such as inflammatory bowel disease (IBD).^[^ ^12^ ^]^ Current diagnostic measures for IBD, including endoscopy and sampling of GI biopsies,^[^ ^13^ ^]^ are invasive and carry substantial healthcare costs. Orally administered bioconjugated nanoparticles, could target inflamed tissue and enable non‐invasive, IBD‐specific imaging and drug delivery. This would be particularly beneficial for pediatric patients who require sedation during endoscopic examinations routinely performed during the diagnosis of IBD.

Despite the key advantages of targeted nanomedicines, they have to date still not made it to clinical use. A major hurdle for the clinical translation of actively targeted nanoparticles is their well‐controlled bioconjugation with targeting moieties. The development of reproducible functionalization protocols is challenged by the unique characteristics of nanoparticles, including their strong agglomeration and sedimentation, and adherence to magnets used for mixing of reagents (for magnetic nanoparticles). Antibody attachment onto the nanoparticle surface can be achieved through various strategies, which are mainly categorized into physical adsorption or covalent binding. The covalent bond can prevent antibody detachment due to changes in pH or competitive displacement by endogenous molecules, such as proteins.^[^ ^9^, ^14^ ^]^

In environments resembling human serum conditions, previous studies have shown that particles can effectively bind to the antigen in cell lysates, regardless of whether ligands are attached covalently or by adsorption.^[^ ^15^ ^]^ However, this is a more constant environment as compared to the dynamic GI tract where pH and enzyme concentrations fluctuate during the digestion of ingested food. Covalently bound antibodies are favorable for orally administered nanoparticles as they remain resistant to detachment or displacement under changing environmental conditions encountered in the GI tract.

Covalently attached antibody‐conjugated nanoparticles have previously been reported, where the most common linkages were via 1) *carbodiimide coupling*, in which amide bonds are formed by crosslinking of carboxylic acids and primary amines using 1‐ethyl‐3‐(3‐dimethylaminopropyl)carbodiimide (EDC) or *N,N*‐dicyclohexylcarbodiimide^[^ ^16^, ^17^, ^18^, ^19^, ^20^ ^]^; 2) *maleimide‐thiol coupling*, which involves the reaction between maleimides and thiols from reduced disulfide bridges of the antibody to yield a thiosuccinimide conjugate^[^ ^18^, ^21^, ^22^, ^23^, ^24^, ^25^, ^26^ ^]^; and 3) *click chemistry*, which consists of multiple regiospecific and high yielding reactions, including the copper‐catalyzed azide‐alkyne cycloaddition (CuAAC)^[^ ^27^, ^28^ ^]^ and the strain‐promoted azide‐alkyne cycloaddition (SPAAC).^[^ ^29^, ^30^, ^31^ ^]^

Conjugating nanoparticles with antibodies, comprising antigen‐binding regions, requires careful consideration of ligand orientation to ensure exposure of the active site for successful target recognition and binding. Given that the carbodiimide reaction involves the conjugation of carboxylic acids and amines, which are prevalent in amino acid side chains distributed abundantly throughout the antibody structure, this reaction fails to offer control over the orientation of antibodies on the particle surface. Likewise, the maleimide reaction is limited by its inability to provide precise control over ligand orientation, while also posing a risk of disrupting the native structure of antibodies, potentially leading to loss of site‐selectivity. In this context, the click reaction presents a distinct advantage over other conjugation strategies by offering precise control over antibody orientation during covalent conjugation. This is achieved by incorporating azide moieties on the heavy chains of an IgG antibody via enzymatic modification.^[^ ^32^ ^]^

CuAAC and SPAAC are widely used for synthesizing complex molecules, such as drug candidates and bioconjugates, with high precision. These reactions play a crucial role in the development of antibody‐drug conjugates, enabling the site‐specific attachment of cytotoxic drugs to antibodies, thus improving the efficacy and safety of targeted cancer therapies.^[^ ^33^, ^34^, ^35^ ^]^ Beyond pharmaceuticals, CuAAC and SPAAC are utilized in materials science for creating functional polymers and nanomaterials, in proteomics for labeling and tracking biomolecules,^[^ ^36^ ^]^ and in diagnostics for developing sensitive assays.^[^ ^37^, ^38^ ^]^ Its versatility and reliability have made click chemistry a cornerstone technique in modern chemical biology and materials science.

**Figure 1:**
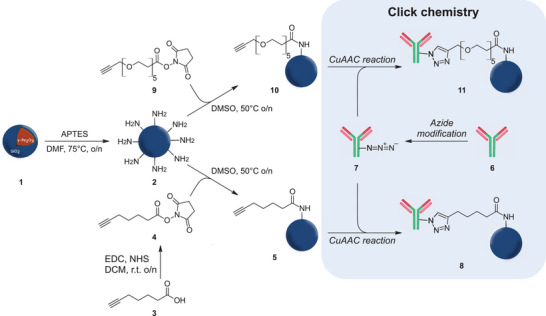
Attachment of antibody ligands (**6**) onto SPION (**1**) surface via click chemistry. Amines were introduced to the SPION surface through a silanization reaction using (3‐aminopropyl)triethoxysilane (APTES) (**2**). An alkyne‐containing linker was synthesized using EDC/NHS crosslinking to yield the NHS‐activated carbonyl compound (**4**). Alkyne moieties were installed onto the nanoparticle surface to yield SPION‐linker (**5**) and SPION‐PEG (**10**), followed by reaction with azide‐modified antibodies (**7**) to yield the final bioconjugated particles SPION‐linker‐Ab (**8**) and SPION‐PEG‐Ab (**11**).

In this study, superparamagnetic iron oxide nanoparticles (SPIONs) that are clinically established contrast agents for MRI,^[^ ^39^, ^40^, ^41^, ^42^, ^43^ ^]^ were functionalized with antibodies targeting upregulated inflammatory biomarkers in the GI tract. In our previous work, biomarkers of inflammation were identified in preclinical IBD‐models, including Intercellular adhesion molecule 1 (ICAM1) and Carcinoembryonic antigen‐related cell adhesion molecule 1 (CEACAM1).^[^ ^44^ ^]^ Here, the aim was to conjugate the constant region of anti‐ICAM1 antibodies onto SPIONs via CuAAC reaction to control the antibody orientation and ensure ligand exposure of its binding region. Each stage of the multi‐step functionalization method was carefully designed and characterized to achieve a highly reproducible protocol. This procedure included successive additions of organic functional moieties that enable the controlled attachment of antibodies onto the SPION surface via click chemistry (**Figure** **1**). The targeting capability of the functionalized SPIONs was investigated on inflammation‐induced Caco‐2 cells. The findings from the in vitro model showed that ICAM1‐conjugation significantly increased the targeting capability of SPIONs compared to the corresponding non‐conjugated particles. In addition, confocal imaging indicated that the particles were internalized by the inflammation‐induced Caco‐2 cells.

## 2 Results and Discussion

The bioconjugation of SPIONs consisted of a multi‐step functionalization procedure, including reactions for silanization, amide formation, and click chemistry (Figure 1). The nanoparticles (**1**) were synthesized and coated with silica (SiO_2_) using flame spray pyrolysis as reported previously in the literature.^[^ ^45^ ^]^ The surface silanol groups serve as a starting point for the functionalization. Post‐synthesis modification of SPIONs involved an initial introduction of amines onto the nanoparticle surface (**2**). This provided the nanoparticles with stronger nucleophilic groups, surpassing the hydroxyl groups of the silica coating, but also enabled the subsequent formation of robust amide linkages, a bond highly resistant to hydrolysis and with reduced susceptibility to nucleophilic attacks compared to other carboxylic acid derivatives. Two different alkyne‐containing compounds: a small organic linker (**4**) and a PEG‐linker (**9**), were conjugated to the SPION surface (**5** and **10**, respectively). The incorporation of PEG aimed to impart hydrophilicity to the nanoparticles, thereby potentially enhancing their colloidal stability in suspension. The alkyne moieties of these compounds served as crucial building blocks to enable the final bioconjugation with azide‐modified antibodies (**7**) via CuAAC chemistry to yield the antibody‐targeted SPIONs (**8** and **11**).

### 2.1 Preparation of ICAM1‐Conjugated SPIONs

#### 2.1.1 Synthesis and Characterization of Iron Oxide Nanoparticles

**Figure 2:**
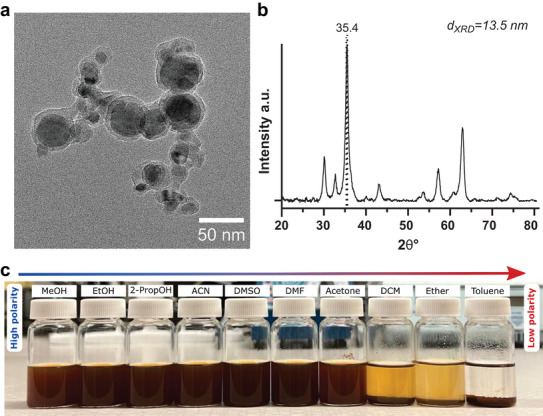
Characterization of SPIONs. TEM micrograph a) and XRD pattern b) of SiO_2_‐coated γ−Fe_2_O_3_ nanoparticles (SPIONs). The dashed line indicates the (311) plane of maghemite. c) Suspensions of SPIONs in various solvents arranged from high to low polarity (left to right). Solvents: Methanol (MeOH), ethanol (EtOH), isopropanol (2‐PropOH), acetonitrile (ACN), dimethylsulfoxide (DMSO), dimethylformamide (DMF), acetone, dichloromethane (DCM), diethyl ether (ether), and toluene.

Particles were produced by flame spray pyrolysis, a manufacturing technique with demonstrated scalability and reproducibility.^[^ ^46^, ^47^ ^]^ This method enables synthesis and in situ silica (SiO_2_) coating of iron oxide (γ‐Fe_2_O_3_) nanoparticles.^[^ ^45^ ^]^ Modifying particles with a SiO_2_ coating is common practice for enhancing the functionality and biocompatibility of magnetic γ‐Fe_2_O_3_ nanoparticles.^[^ ^48^, ^49^ ^]^ Specifically, the silica coating greatly enhances nanoparticle dispersion in aqueous media and prevents strong agglomeration observed for uncoated γ‐Fe_2_O_3_.^[^ ^49^ ^]^ The one‐step production method yielded SiO_2_‐coated γ−Fe_2_O_3_ nanoparticles, as visualized with transmission electron microscopy (TEM) (**Figure** **2**a). In agreement with previously reported literature,^[^ ^45^ ^]^ the X‐ray diffraction (XRD) pattern indicates that the particles are γ‐Fe_2_O_3_ (maghemite) with the (311) plane at 2θ = 35.4° (Figure 2b). The crystallite size was 13.5 nm and thus smaller than d_TEM_ (17.6 ± 1.4 nm, Figure S1, Supporting Information) indicating polycrystalline particles. The geometric standard deviation (σ_g_) of the primary particle size distribution (Figure S1, Supporting Information) was σ_g_ = 1.4 in good agreement with the self‐preserving size distribution consistently obtained for flame‐made nanoparticles.^[^ ^45^ ^]^ Overall, these flame‐made SiO_2_‐coated γ−Fe_2_O_3_ particles are in the superparamagnetic domain. The larger particle fraction in their size distribution is ferrimagnetic and thus introduces a slight hysteresis in the magnetization curves.^[^ ^50^ ^]^ Nevertheless, the coercivity is low,^[^ ^45^ ^]^ and flame‐made nanoparticles have been reported to exhibit excellent T2 contrast enhancement in MRI.^[^ ^51^, ^52^ ^]^ These SiO_2_‐coated γ−Fe_2_O_3_ particles are referred to as SPIONs from now on.

#### 2.1.2 Conjugation of (3‐aminopropyl)triethoxysilane

Solvent choice plays a crucial role during the functionalization of nanoparticles to ensure dispersion and avoid agglomeration during conjugation reactions. The vast majority of current literature, reports the conjugation of (3‐aminopropyl)triethoxysilane (APTES) onto SiO_2_ surfaces in solvents such as toluene, ethanol, methanol, or mixtures thereof.^[^ ^53^, ^54^, ^55^ ^]^ Dispersion of flame‐made SPIONs was investigated in various organic solvents to find suitable reaction conditions for the APTES conjugation. Particle suspensions of 1 mg/mL were prepared by dispersing the particles in methanol (MeOH), ethanol (EtOH), isopropanol (2‐PropOH), acetonitrile (ACN), dimethylsulfoxide (DMSO), dimethylformamide (DMF), acetone, dichloromethane (DCM), diethyl ether (ether) and toluene. Visual assessment of the suspensions confirmed that the particles agglomerated and sedimented quickly when suspended in DCM, ether, or toluene. This makes them the least suitable solvents for any modification reaction, while the more polar ones seemed to disperse the particles. These results are expected as the hydroxyl‐rich surface of the SiO_2_‐coated particles should make them easily dispersed in polar solvents rather than in less polar ones (Figure 2c). With this reasoning, EtOH would be the optimal solvent for surface modification of SPIONs. While this may be true for certain reactions, it was not suitable for the APTES‐conjugation of the SPIONs here. Attenuated total reflectance Fourier transform infrared (ATR‐FTIR) spectroscopy showed none of the characteristic functional moieties of APTES for particles undergoing APTES‐reaction in EtOH (Figure S2a, Supporting Information). Similarly, thermogravimetric analysis (TGA) did not detect any weight reduction compared to the pristine particles (Figure S2b, Supporting Information).

**Figure 3:**
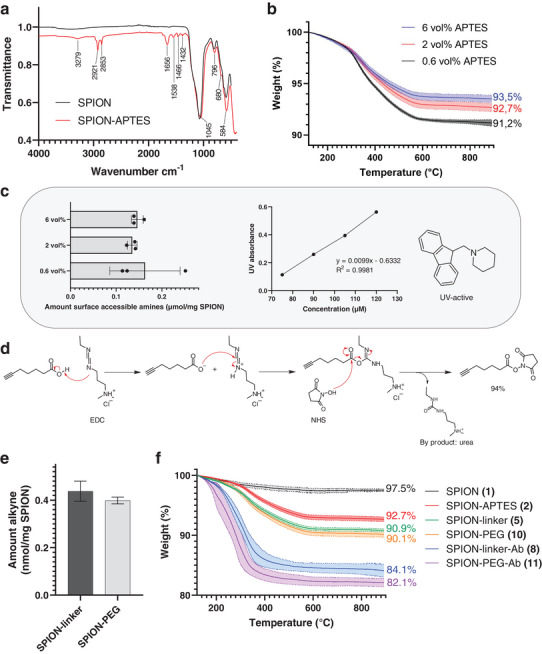
Characterization of functionalized SPIONs. a) ATR‐FTIR spectra of APTES‐modified SPIONs. b) TGA profiles of SPIONs functionalized with 0.6 vol%, 2 vol%, and 6 vol% APTES (n ≥ 5). c) UV‐based quantification of dibenzofulvene‐adduct corresponding to surface accessible amine groups present on SPION surface, values normalized to µmol/mg of SPION (n = 3). d) Reaction mechanism for synthesis of small organic linker using EDC‐NHS cross‐coupling. e) Quantification of alkynes on particle surface using fluorescent click‐labeling, values normalized to nmol/mg of SPION (n = 3). f) TGA profiles representing mean and standard deviation of products obtained after indicated modification step, values normalized to 120 °C (n ≥ 3).

The general reaction mechanism for SiO_2_ functionalization with APTES includes initial hydrolysis of the ethoxy groups, followed by self‐condensation of APTES molecules producing siloxane (Si─O─Si) bonds and condensation with the substrate.^[^ ^56^ ^]^ Numerous reaction parameters have been reported to covalently link APTES to different substrate surfaces in order to achieve monolayers, multilayers, various thicknesses, and densities of APTES.^[^ ^54^, ^57^, ^58^, ^59^, ^60^ ^]^ Curing the substrate at elevated temperatures has generally been assumed to enhance the horizontal polymerization of APTES, however, studies have shown that the actual reaction temperature may also play a significant role.^[^ ^58^, ^61^ ^]^ Therefore, the APTES‐reaction was performed in DMF (bp: 153 °C), which also efficiently dispersed the particles, but had a much higher boiling point than EtOH (bp: 78 °C). Thus, the reaction can be performed at higher temperatures using DMF as solvent. Successful APTES‐functionalization was achieved using DMF as the solvent and a temperature of 75 °C, as confirmed by ATR‐FTIR spectroscopy (**Figure** **3**a). The bands observed at 2921 and 2853 cm^−1^ were assigned to the stretching modes of CH_2_.^[^ ^62^ ^]^ The bands at 1566 and 1438 cm^−1^ were ascribed to NH_2_ deformation modes of the amine group,^[^ ^62^ ^]^ and the broad band at 3279 cm^−1^ was attributed to the N─H stretching vibration, confirming the presence of amine functional groups on the SPION surface. The band observed ≈1432 cm^−1^ was assigned to Si─C stretching, and bands at 1045 and 796 cm^−1^ are due to Si─O─Si and Si─OH vibrations from the SiO_2_ coating of the particles. The successful conjugation of APTES was further confirmed by TGA analysis (Figure 3f), which showed a larger weight loss (92.7%) compared to the non‐conjugated SPIONs (97.5%). Using the count mean diameter d_TEM_ = 17.6 nm and a density of ρ = 4.28 g cm^−^^3^ for SiO₂‐coated γ‐Fe₂O₃, this weight reduction corresponds to ≈1.6 APTES‐molecules nm^−2^. The 2.5% weight loss of the pristine particles is common for flame‐made nanomaterials and can be attributed to non‐combusted precursor material and/or adventitious carbon. Furthermore, a slight decrease in the hydrodynamic size of APTES‐modified SPIONs in DMSO (146.2 nm) was observed compared to the pristine SPIONs in DMF (193.7 nm) (**Table** **1**). However, this minor variation is more likely attributed to the differing dispersibility properties resulting from the use of various solvents rather than differences in the surface chemistry of the particles. On the other hand, the zeta potential of APTES‐modified SPIONs was ζ = +33.5 mV, which is a considerable change from the pristine SPIONs with a zeta potential ζ = −30.3 mV, suggesting successful APTES‐conjugation (Table 1). Although DMF was selected as the optimal solvent for the APTES‐conjugation, exploring other more sustainable alternatives is important. The use of DMF is restricted by the European Chemicals Agency's Registration, Evaluation, Authorization, and Restriction of Chemicals (REACH).^[^ ^63^ ^]^ DMF is a widely used solvent in various chemical and pharmaceutical processes, but its environmental and health hazards underscore the importance of exploring more eco‐friendly alternatives to promote sustainable and safer practices. While EtOH was found to be inefficient for the APTES‐conjugation here, other solvents similar to DMF such as propylene carbonate, a polar aprotic solvent with a high boiling point, warrant further investigation.

**Table 1:** Hydrodynamic size, polydispersity index (PDI), and zeta potential of functionalized SPIONs in various solvents (n = 3).

Functionalized particle	Hydrodynamic diameter [nm]	PDI [%]	Zeta potential [mV]	Solvent
SPION (1)	193.7 ± 0.83	21.4	−30.3 ± 5.6	DMF
SPION‐APTES (2)	146.2 ± 1.6	18.4	33.5 ± 1.2	DMSO
SPION‐linker (5)	153.0 ± 0.9	20.0	24.2 ± 3.2	H_2_O
SPION‐PEG (10)	159.6 ± 3.3	20.2	25.0 ± 2.3	H_2_O
SPION‐linker‐Ab (8)	>1000^a)^	27.5	10.7^*^	H_2_O
SPION‐PEG‐Ab (11)	>1000^a)^	24.0	7.9^*^	H_2_O

^a)^ Measured in one replicate.

Optimizing the amount of APTES on the surface of the SPIONs is of importance as the following functionalization reaction is dependent on the presence of amines on the particles. Varying the concentration of APTES in the reaction will affect the amount and the thickness of the APTES‐layer on the SPION surface, however, it may not necessarily enhance the amount of surface‐accessible amines. A concentration of 2 vol% has been frequently used for grafting APTES onto oxide surfaces.^[^ ^54^, ^55^, ^57^, ^61^ ^]^ Therefore, in order to understand if the added APTES concentration affects the amount of surface accessible amines, particles conjugated with higher (6 vol%) and lower (0.6 vol%) concentrations of APTES were produced and characterized. In this study, the TGA profiles of the particles showed the inverse correlation of larger weight loss with lower concentration of APTES added to the reaction mixture (Figure 3b). These results are quite surprising as they indicate that the weight loss does not correlate with the amount of added organic material in the reaction mixture. A possible explanation is that DMF is hygroscopic and its water content may induce rapid hydrolysis and polymerization of APTES. The polymerization may be more significant when adding larger volumes of APTES due to high local concentration in the reaction mixture, leading to its subsequent inefficient grafting onto the particles. However, as APTES tends to form complex networks of linkages and interactions with itself and the surfaces it is grafted on, a larger amount of APTES on the particles may not necessarily enhance the amount of surface‐accessible amines.

To determine the number of amine groups on APTES‐modified SPIONs, the particles were reacted with 9‐fluorenylmethoxycarbonyl chloride (Fmoc‐Cl), commonly used to prevent undesirable side reactions during peptide synthesis.^[^ ^64^ ^]^ Quantification of surface accessible amines was performed by attaching the Fmoc‐groups to the free amines of the particles. The Fmoc‐group was then cleaved using the base piperidine, which in turn produces the UV‐active compound dibenzofulvene‐piperidine adduct (Figure S3, Supporting Information) that is easily quantified using UV–vis spectroscopy. The amount of dibenzofulvene adduct in the reaction mixture thus enables the estimation of the number of amine groups present on the SPION surface. The maximum absorption at 301 nm was measured and quantified by comparison of the UV absorbance to a calibration curve that was prepared by measuring the absorbance of Fmoc‐Valine‐OH at varying concentrations (µM). This curve showed a linear correlation (y = 0.0099x – 0.6332; R^2^ = 0.9981) (Figure 3c). Despite the almost three to ten fold difference of added APTES in the different reactions, the estimated concentrations of amine groups did not differ significantly between the various APTES‐modified particles. The variability between batches was larger in the 0.6 vol% APTES‐reaction, however, without a significant variation of the average values, suggesting that increased quantities of APTES on SPION surfaces do not necessarily correspond to their more extensive functionalization for further modification and biochemical use.

#### 2.1.3 Installing an Alkyne Handle for Click Reaction

A functional handle has to be added to the SPION surface to enable antibody attachment via click chemistry. Thus, two types of organic linkers were added to produce particles with slightly different surface chemistry: *(i)* a small, flexible, and easy‐to‐make organic linker containing an *N*‐hydroxysuccinimide (NHS)‐ester and a terminal alkyne (Figure 1, **compound 4**), referred to as linker, and *(ii)* a slightly larger, polyether containing compound that also comprises an NHS‐ester and a terminal alkyne (Figure 1, **compound 9**), referred to as PEG.

The NHS‐activated carbonyl carbon in both linkers is susceptible to nucleophilic attack by the amine groups on the SPIONs, making the installment of the linker and PEG onto the particle surface rather straightforward. The first linker was synthesized via EDC/NHS crosslinking (94% yield) (Figure 3d, Figure S4, Supporting Information), whereas the PEG‐linker was commercially available. Successful linker‐ and PEG‐conjugation was confirmed with TGA (Figure 3f), showing a moderately reduced weight loss of linker‐modified (90.9%) and PEG‐modified (90.1%) SPIONs compared to the APTES‐modified ones (92.7%). Based on these weight losses, the number of linkers and PEG on the SPION surface was estimated to be 0.61 and 0.50 molecules nm^−2^, respectively. The hydrodynamic size remained rather unchanged after the conjugations (Table 1), however, the zeta potential was reduced from ζ = 33.5 mV of the previous step, to ζ = 24.2 mV and ζ = 25.0 mV for linker‐ and PEG‐conjugated particles, respectively (Table 1). The polydispersity index (PDI) was ≈20% for as‐synthesized and functionalized SPIONs, indicating a narrow size distribution (Table 1).

The small linker might limit the dispersibility of the particles due to the hydrophobic nature of aliphatic carbon moieties. Based on the hydrodynamic size and zeta potential, PEG did not improve the hydrophilic properties of the particles compared to the small linker‐conjugated SPIONs. The number of alkynes present on the SPION surface was quantified by clicking the particles with an azide‐containing fluorophore and quantifying the fluorescence intensity in each sample (Figure 3e). A calibration curve was prepared using a 488‐azide‐containing fluorophore (861.04 g mol^−1^) versus fluorescence, demonstrating a linear correlation (y = 982.0x–312.0; R^2^ = 0.9978) (Figure S5, Supporting Information). The linker‐ and PEG‐conjugated particles were estimated to have 0.45 µmol/mg (± 0.03 µmol) and 0.41 µmol alkynes mg^−1^ SPIONs (±0.01 µmol), respectively. Although the small linker was added at almost double equivalence to PEG, approximately the same number of alkynes were estimated on the SPION surface, which could be attributed to the number of surface accessible amines, which are equal prior to the linker‐ or PEG‐conjugation (Figure 3c). Images of the fluorescently labeled linker‐ and PEG‐conjugated particles were obtained with fluorescence microscopy, visually confirming the presence of the linker and PEG on the nanoparticles (Figure S6, Supporting Information).

#### 2.1.4 Clicking Together Anti‐ICAM1 Antibodies and SPIONs

The successful click conjugation between SPIONs and antibodies requires the presence of alkynes on one reactant and azides on the other. As alkynes were introduced onto the SPIONs as described above, the antibodies, which typically lack azide functionalities, were structurally modified to present azides. This was accomplished by using the Invitrogen SiteClick Antibody Azido Modification kit, following the experimental protocol provided by the manufacturer. This well‐established protocol is frequently used in literature to enzymatically attach azide tags to carbohydrate groups on the antibody, resulting in azide modification specifically in the Fc region.^[^ ^65^, ^66^ ^]^ Modifications within the Fc‐region, the constant domain of the immunoglobulin G (IgG) antibody, offer a facile route to tailor the targeting specificity, thereby enabling interchangeability with other IgG antibodies of interest. This strategic modification not only facilitates the conjugation process but also broadens the applicability of the platform to diverse antibody targets, highlighting its versatility in targeting applications.

Important targets for disease management and diagnosis include elevated levels of biomarkers. ICAM1 is a cell surface glycoprotein expressed by various cell types. These include leukocytes and endothelial cells known to drive inflammatory responses in various conditions, such as lung inflammation, atherosclerosis, and IBD.^[^ ^44^, ^67^, ^68^ ^]^ Therefore, anti‐ICAM1 antibodies were prepared for the click‐conjugation by azide‐modification of the Fc‐region (Figure 1, **compound 7**). These were then conjugated to alkyne‐modified SPIONs via CuAAC reaction. This click reaction depends on the presence of a copper catalyst, facilitating the formation of the triazole ring between the SPION and the antibody. Successful antibody‐conjugation was indicated by a reduction of the zeta‐potential to ζ = 10.7 mV and ζ = 7.9 mV for linker‐Ab‐ and PEG‐Ab‐conjugated particles, respectively. This near‐neutral zeta potential also resulted in rapid aggregation of the bioconjugated particles in water as measured by their large (> 1000 nm) hydrodynamic diameter (Table 1). Although inducing sedimentation, the near‐neutral charge is considered advantageous for mucus penetration and intestinal drug delivery of nanoparticle‐based systems.^[^ ^69^, ^70^ ^]^

**Figure 4:**
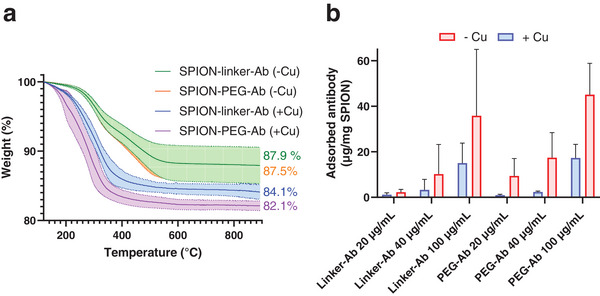
Characterization of SPIONs antibody click‐conjugated in the presence (+Cu) and absence of copper (Cu). a) TGA profiles of particles prepared with 100 µg mL^−1^ antibodies (n ≥ 2). b) The adsorbed antibody as determined by SDS‐PAGE on SPIONs conjugated with various amounts of antibody (20–100 µg mL^−1^) in the presence (+Cu, blue bars) and absence (‐Cu, red bars) of copper catalyst (n = 3).

The amount of antibodies bound to the particles was quantified by back‐calculating the concentration of excess antibodies in the reaction mixture using the Bicinchoninic Acid (BCA) assay (Figure S7, Supporting Information). Particles conjugated with antibodies in the absence of copper were used as a control. The results suggested comparable antibody quantities on the particles across all reactions, regardless of the presence of the copper catalyst. However, TGA profiles revealed a greater weight reduction for SPION‐linker‐Ab (84.1%) and SPION‐PEG‐Ab (82.1%) compared to the corresponding copper‐free reactions (87.9% and 87.5%, respectively) (**Figure** **4**a). Additionally, the copper‐free antibody conjugations resulted in increased variability between batches. Thus, despite the azide‐alkyne cycloaddition requiring copper catalysis for fast reactions at mild conditions,^[^ ^71^, ^72^ ^]^ the antibodies evidently adhered to the SPIONs even in the absence of a catalyst. This phenomenon could be attributed to the formation of a biomolecular corona on the particle surface. This adsorption of antibodies may also account for the slightly increased PDI values for the SPION‐linker‐Ab (27.5%) and SPION‐PEG‐Ab (24%) (Table 1). However, as these PDI values are < 30%, they can still be deemed acceptable for clinical application and comparable to those measured for other nanocarriers.^[^ ^73^ ^]^

Adsorbed antibody on the SPION surface was quantified by SDS‐PAGE. Particles conjugated with varying amounts of antibody (20–100 µg mL^−1^), with and without copper catalyst were analyzed (Figure 4b). In the presence of copper, less intense bands were observed on the gel, corresponding to fewer adsorbed antibodies, compared to the reactions that proceeded without catalyst. The degree of adsorption increased with increasing antibody concentration. There was a clear trend of less adsorbed antibodies on particles bioconjugated with copper at all antibody concentrations tested (Figure 4b; Table S2, Supporting Information), although not statistically significant. The lack of statistical significance could be attributed to a large variability between the batches for the copper‐free reactions, as confirmed also by TGA (Figure 4a). This suggests that the adsorption of antibodies on the particle surface is an uncontrolled and highly variable process. The estimated number of adsorbed antibodies per nanoparticle was 0.7 (+Cu) and 1.7 (‐Cu) for SPION‐linker‐Ab, and 0.8 (+Cu) and 2.2 (Cu) for SPION‐PEG‐Ab, all prepared with 100 µg mL^−1^ antibodies in the reaction mixture. Thus, in the absence of copper, the number of adsorbed antibodies increases by a factor of 2.4–2.8. The corona formation could be mitigated by employing PEG of varying lengths. However, the PEG‐molecule utilized in this study may be too short to effectively prevent undesirable antibody adherence. Thus, enhancing the hydrophilicity by introducing charged moieties or longer PEG‐chains to the SPION surface and limiting the quantity of added antibodies (Figure 4b) may offer means to regulate and control corona formation.

To estimate the number of covalently attached antibodies per nanoparticle, the difference between the TGA‐based weight loss of SPIONs conjugated in the absence and the presence of copper was used (Figure 4a). The number of antibodies (calculated as detailed in the Supporting Information) was ≈1.8 antibodies/nanoparticle for SPION‐linker‐Ab (weight reduction of 3.8 ± 1.9%) and 2.6 antibodies/nanoparticle for SPION‐PEG‐Ab (weight reduction of 5.4 ± 1.3%). Thus, there is a 2.6 to 3.3 times greater amount of covalently bound antibodies than adsorbed ones on the nanoparticle surface. Overall, these results suggest that a large fraction of the antibodies are covalently bound to the SPIONs after CuAAC bioconjugation. The number of targeting ligands conjugated to each nanoparticle is determined by several factors, including the nanoparticle size, specific surface area, choice of ligand, and the reaction conditions and chemical linkages used to introduce reactive functional groups onto the nanoparticle surface. Consequently, the surface loading of antibodies can vary significantly across different studies and their targeting efficacy has to be assessed in relevant preclinical models for the intended application.

### 2.2 Targeting Capability of Anti‐ICAM1‐Conjugated SPIONs

#### 2.2.1 Cell Viability after Exposure to SPIONs

Investigation of cell viability after SPION exposure is important for evaluating their biocompatibility and potential use for biomedical applications. The cell viability of Caco‐2 cells after exposure to bare γ−Fe_2_O_3_ has previously been explored,^[^ ^74^ ^]^ showing no adverse effects across a range of concentrations. However, the introduction of a targeting moiety on the SPION surface could alter particle‐cell interactions, thereby raising concerns regarding toxicity.

As undifferentiated Caco‐2 cells exhibit greater sensitivity to toxicity compared to their differentiated counterparts,^[^ ^75^ ^]^ cell viability tests using undifferentiated Caco‐2 cells provide an estimation of the worst‐case scenario. In this study, four types of SPIONs were investigated: bare γ−Fe_2_O_3_, SiO_2_‐coated γ−Fe_2_O_3_ (SPION), SPION‐linker‐Ab, and SPION‐PEG‐Ab particles. The antibodies themselves can exert toxicity, therefore, the particles were prepared by adding 100 µg mL^−1^ antibody to the reaction mixture, which is the highest concentration investigated in this study.

**Figure 5:**
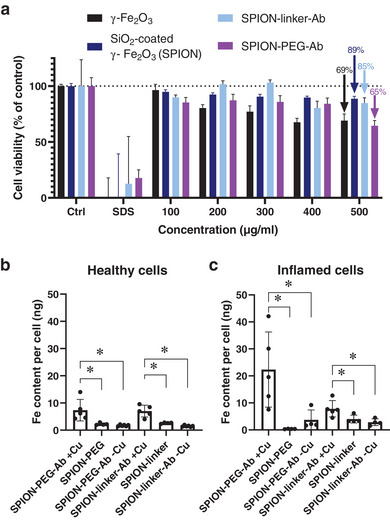
Functionalized SPION exposure to Caco‐2 cell monolayers. a) Cell viability of undifferentiated Caco‐2 cells after exposure to functionalized SPIONs at different particle concentrations (100–500 µg mL^−1^) for 24 h. Cell viability was determined using the CellTiter‐Glo luminescent cell viability assay and calculated as a percentage of the control (set to 100%) (n = 6). ICP‐OES‐based quantification of Fe in healthy b) and inflamed c) cell monolayers after exposure to SPIONs for 2 h (n ≥ 4). Differences were calculated by *t*‐test (*p < 0.05).

The cell viability was high even after exposure to the highest particle concentration of 500 µg mL^−1^ (**Figure** **5**a). Notably, the SiO_2_ coating demonstrated a significant enhancement in the cytocompatibility of the particles, as evidenced by a cell viability of 89% following exposure to the highest investigated dose. Cells exposed to SPION‐PEG‐Ab exhibited a moderate decrease in viability, with a survival rate of 65% observed at the highest exposure dose. This finding aligns with literature reports indicating that shorter PEG‐chains tend to exert a cytotoxic effect on cells.^[^ ^76^, ^77^, ^78^ ^]^ On the other hand, cells exposed to SPION‐linker‐Ab maintained a viability rate of 85% at the highest exposure dose. Therefore, the small linker offers the advantage of cost‐effectiveness, enhanced biocompatibility, and facile synthesis in laboratory settings without the need for extensive purification. Overall, nanoparticle exposure for all studied particles was well tolerated up to a concentration of 400 µg mL^−1^, with cell viability exceeding 70%. This surpasses the non‐toxicity threshold specified in the international standard ISO 10993–5.^[^ ^79^ ^]^ Even at the highest exposure dose of 500 µg mL^−1^, SPIONs and SPION‐linker‐Ab maintained acceptable cell viability levels.

These findings underscore the importance of surface modifications in modulating the cytotoxicity of SPIONs. Despite the cells being undifferentiated and thus more sensitive to toxic events, the high viability observed even at the highest exposure doses demonstrates the suitability of the functionalized SPIONs for biomedical use, even though further studies on the underlying mechanisms of cytotoxicity are warranted to ensure their safe utilization.

#### 2.2.2 Binding Efficacy of Antibody‐Conjugated SPIONs

Nanoparticle targeting and internalization are highly influenced by the chosen cell model, specific cellular target, culture conditions, and the duration of incubation with the nanoparticles. These variables make it challenging to directly compare the targeting efficiency of different nanoparticle systems. Here, the binding efficacy of the bioconjugated SPIONs was assessed using inflammation‐induced Caco‐2 cells, grown on Transwell filters. In assessing their targeting efficacy, a critical challenge lies in the risk of nanoparticle sedimentation on the cell surface during exposure, which could lead to false positive results. To overcome this issue and accurately evaluate the binding efficacy of antibody‐conjugated SPIONs, Caco‐2 cells were seeded on the undersurface of Transwell filters until fully differentiated. Inflammation was then induced on the basolateral side of the cells by a mixture of cytokines (tumor necrosis factor‐α, interleukin‐1β, lipopolysaccharides, and interferon‐γ).^[^ ^80^ ^]^ Trans‐epithelial electrical resistance (TEER) measurements were conducted (Table S3, Supporting Information) to confirm inflammation of the monolayers, as evidenced by a decrease in overall TEER values, indicating compromised barrier integrity.

**Figure 6:**
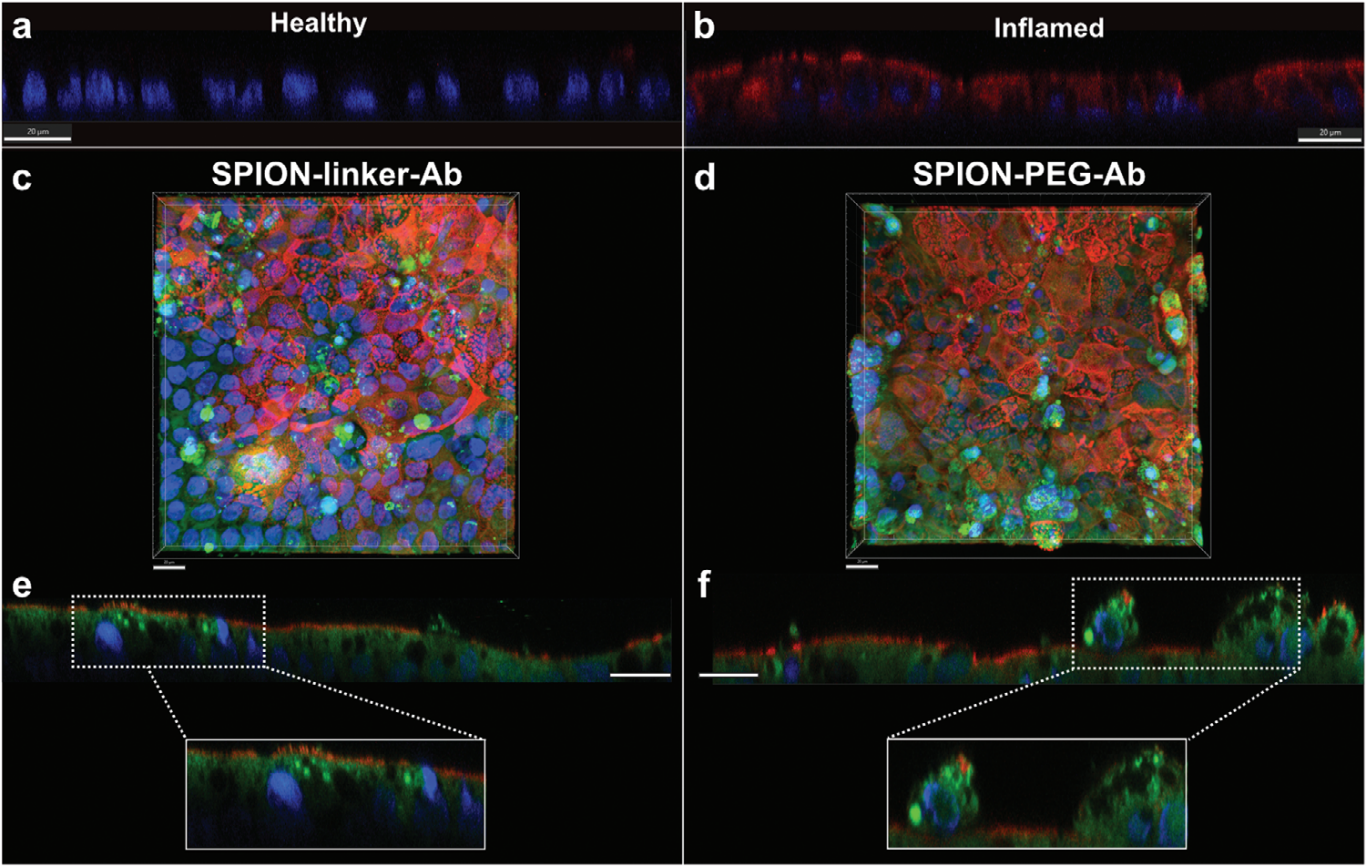
Confocal images of differentiated and inflammation‐induced Caco‐2 cell monolayers cultured in Transwell filters and stained for ICAM1 (red) and nuclei (blue). Cross‐sectional images of healthy a) and inflammation‐induced b) cells. Overview c,d) and cross‐sectional e,f) images of inflamed cells exposed to functionalized SPIONs (green) (scale bar: 20 µm).

Inductively coupled plasma‐optical emission spectrometry (ICP‐OES) was employed to quantify the concentration of iron (Fe) in healthy and inflamed Caco‐2 cells following a 2 h exposure to ICAM1‐targeted (prepared with 100 µg mL^−1^ antibody) and non‐targeted SPIONs. The 2 h exposure time was selected as it is a well‐established and tolerated duration for Caco‐2 cells under these culturing conditions.^[^ ^81^ ^]^ Overall, higher concentrations of Fe were measured in inflamed cells compared to healthy counterparts, particularly evident in cells exposed to SPION‐PEG‐Ab (Figure 5b,c). However, despite this trend, a statistically significant difference between the healthy and inflamed conditions did not emerge. This observation highlights the inherent challenges associated with the identification of an ideal IBD‐specific biomarker. Healthy and inflammation‐induced cells were stained for ICAM1 (red) and nuclei (blue), and visualized using confocal microscopy to showcase the ICAM1 expression in the two models (**Figure** **6**a,b). ICAM1 was present in both healthy and inflamed cells, but a clear upregulation of ICAM1 was observed in the inflamed state in agreement with our previous findings with proteomics‐based quantification of ICAM1.^[^ ^44^ ^]^

Importantly, a significantly elevated concentration of Fe was observed in cells exposed to antibody‐conjugated SPIONs compared to their non‐conjugated counterparts (Figure 5b,c). This significant difference between conjugated and non‐conjugated particles emphasizes the efficacy of the ligand in enhancing the targeting capability of the SPIONs. Comparative assessment of Fe content in cells exposed to SPION‐linker‐Ab and SPION‐PEG‐Ab suggests a potential advantage of the latter particles in the inflamed cell model. While previous reports on the influence of PEG‐linkers on cellular uptake have yielded conflicting results,^[^ ^82^, ^83^, ^84^, ^85^ ^]^ our findings may be attributed to the ability of PEG‐linkers to minimize particle surface interactions with salts and molecules present in the medium. This, in turn, may enhance the specificity of cellular interactions between the targeting antibody and the ICAM1 biomarker. Furthermore, the SPION‐PEG‐Ab had a larger number of covalently bound antibodies on their surface compared to SPION‐linker‐AB (Figure 4a), which might also enhance targeting efficacy.

Moreover, given that the copper‐free conjugation of antibodies led to the formation of a biomolecular corona, it is crucial to determine whether the covalent attachment of antibodies enhances the targeting capability of SPIONs, or if antibodies adsorbed onto the particle surface through non‐covalent interactions are sufficient. To investigate this, cells were exposed to SPIONs bioconjugated under copper‐free conditions and analyzed using ICP‐OES (Figure 5b,c). The findings reveal that the targeting ability is highly dependent on the covalent attachment of antibodies to the particles. Copper‐free bioconjugated particles exhibited poor targeting capacity, similar to particles lacking any targeting ligands on their surface (Figure 5b,c). This suggests that the biomolecular corona may be detaching in the cell culture medium, or that the ligands, due to their uncontrolled orientation, are not optimally positioned to effectively interact with the target.

To further study the interactions of antibody‐conjugated particles with the epithelial cells, inflammation‐induced Caco‐2 cell monolayers were exposed to fluorescently tagged ICAM1‐conjugated SPIONs (prepared with 100 µg mL^−1^ antibody) and visualized using confocal microscopy (Figure 6c–f). The inverted configuration of growing cells on the bottom side of the Transwell membrane eliminates the possibility of sedimentation‐driven particle interaction with the cell surface. Nevertheless, despite the inverted setup, large particle aggregates were observed to adhere to the cell surface, suggesting active binding (Figure 6c,d). In cross‐sectional images, the functionalized SPIONs (visualized in green) were observed both apically and internally within the cells. This observation strongly suggests the internalization of functionalized SPIONs, demonstrating the potential for cellular uptake, which warrants additional investigations to reveal particle uptake mechanisms. Chemical endocytosis inhibitors can be incorporated to selectively obstruct specific cellular uptake pathways and enable the identification of the primary uptake mechanism of the studied system.^[^ ^86^ ^]^ The uptake mechanism of polymeric particles conjugated with ICAM1 in Caco‐2 cells was previously addressed by inhibiting clathrin‐, caveolar‐, and cellular adhesion molecule (CAM)‐mediated endocytosis, and revealed the involvement of predominantly CAM‐mediated pathways.^[^ ^87^ ^]^

The fluorophore‐conjugation to the antibodies served two crucial purposes: first, to confirm the presence of targeting ligands on the SPIONs, and second, to facilitate imaging of the particles to which they are bound. In addition to observing the localization of functionalized SPIONs within the cells, a diffuse green signal is noticeable just beneath the cell membrane (Figure 6e,f). This observation could originate from the presence of adsorbed antibodies on the SPIONs (Figure 4a,b), which could have detached from the particle surface and penetrated the cell membrane. To investigate this, inflamed cells were exposed to fluorescently‐labeled free antibodies for 1 and 2 h (Figure S8a,b, Supporting Information). The green signal was not observed inside the cells, but only on unhealthy cells detaching from the cell monolayer (Figure S8a, Supporting Information). This indicates that the vague green signal observed inside the cells stems from the antibodies conjugated to a nanoparticle prior to cell internalization and not from the uptake of detached, free antibodies. Furthermore, bioconjugated SPIONs prepared with a lower amount of antibody (20 µg mL^−1^) exhibited a similar green signal from inside the cells (Figure S8c–f, Supporting Information). As these particles have a considerably lower amount of adsorbed antibodies on their surface (Figure 4b), this further suggests that the green signal inside the cells primarily arises from covalently attached antibodies.

Overall, the observations from confocal microscopy, when considered alongside the findings from ICP‐OES analysis, strongly indicate the internalization of functionalized SPIONs and the successful binding of antibody‐conjugated SPIONs to the target cells. The covalent attachment of antibodies via click chemistry is crucial for effective targeting and cellular uptake. Nanoparticles with only adsorbed antibodies did not increase cellular uptake compared to their non‐targeted counterparts. The amount of adsorbed antibody could be minimized by fine‐tuning the amount of antibody added to the reaction mixture. While this is of importance to reduce costs during manufacturing of targeted nanoparticles, the targeting efficacy was not impeded by the presence of adsorbed antibodies on click‐conjugated nanoparticles. Furthermore, despite their large size (> 1 µm, Table 1) the particles demonstrated the ability to interact with cells and be internalized. However, if necessary, the surface chemistry can be further optimized to achieve smaller particle sizes for antibody functionalization.

## 3 Conclusion

This study presents a comprehensive investigation of the preparation and functionalization of ICAM1‐conjugated superparamagnetic iron oxide nanoparticles (SPIONs). We synthesized SiO_2_‐coated γ‐Fe_2_O_3_ particles through FSP. The conjugation of APTES onto the SiO_2_‐coated SPIONs was optimized by selecting suitable reaction conditions, and successful functionalization was confirmed using ATR‐FTIR spectroscopy and TGA. Next, an alkyne handle was installed onto the SPION surface for subsequent antibody attachment via click chemistry. Two types of organic linkers were utilized, a small organic linker and PEG, and their conjugation to the SPIONs was validated using TGA. The number of alkynes present on the SPION surface was quantified and comparable results were observed for both linker‐ and PEG‐conjugated SPIONs. It is worth noting that the small linker employed in this study is cheap and easy to synthesize, making it a cost‐effective and convenient option for functionalizing SPION surfaces. Subsequently, anti‐ICAM1 antibodies were conjugated to the alkyne‐modified SPIONs via copper‐catalyzed click chemistry, and successful covalent binding was confirmed through DLS, TGA, and SDS‐PAGE. The silica‐coated nanoparticle surface employed here as a starting point makes the developed functionalization protocol also widely applicable to other types of nanoparticles apart from SPIONs.

Cell viability assays were conducted on undifferentiated Caco‐2 cells and high viability was observed, especially for SiO_2_‐coated and linker‐conjugated SPIONs, compared to uncoated and PEG‐conjugated particles. Moreover, quantitative assessment using ICP‐OES revealed higher iron concentrations in cells exposed to antibody‐conjugated SPIONs compared to non‐conjugated ones in both healthy and inflamed cell models. Confocal microscopy showed evidence of both surface binding and internalization of ICAM1‐conjugated SPIONs by the inflamed Caco‐2 cells. In addition, particles with only adsorbed antibodies were prepared by performing the click reaction in the absence of copper. These particles were not internalized by the inflamed cells, as evidenced by ICP‐OES, highlighting the importance of covalently bound targeting ligands for effective targeting and cellular uptake here. Overall, our findings highlight the potential of ICAM1‐conjugated SPIONs for targeted imaging and therapeutic applications, paving the way for further exploration and development of biomedical nanotechnology for diagnostic applications of inflamed intestinal tissue.

## 4 Experimental Section

### Preparation of SPIONs (product **1**)

SiO_2_‐coated iron oxide nanoparticles were synthesized in an enclosed FSP reactor as described in previous studies.^[^ ^45^ ^]^ A precursor solution of Fe was prepared by dissolving iron(III) acetate (Sigma–Aldrich, Sweden) in a mixture of xylene (Sigma–Aldrich, Sweden) and acetonitrile (Sigma–Aldrich, Sweden) (ratio 3:1) to obtain a total metal concentration of 0.34 m Fe. The precursor solution was stirred for 1 h at room temperature and then fed at 5 mL min^−1^ to the innermost FSP nozzle capillary and dispersed by 5 L min^−1^ O_2_ (>99.5%, Linde AGA Gas AB, Sweden) from the surrounding annulus. The solution spray was ignited by a premixed ring‐shaped flame of methane/oxygen at 1.5/3.2 L min^−1^ (total 4.7 L min^−1^). The pressure drop at the nozzle tip was maintained at 1.5 bar. The FSP reactor was enclosed by a 20 cm long quartz glass tube and the spray flame was sheathed by 40 L min^−1^ O_2_ flowing through the outermost sinter metal plate of the FSP burner. A stainless‐steel metal torus pipe ring with 16 radial equispaced openings was placed on top of the glass tube. Finally, another 30 cm long quartz tube was positioned on top of the torus ring. Hexamethyldisiloxane (HMDSO, Sigma–Aldrich) vapor was introduced to the reactor system through the torus ring, from a bubbler at 10 °C, and mixed with an additional flow of N_2_ gas at 15 L min^−1^. The SiO_2_ content was kept to 23 wt.% by controlling the N_2_ flow rate through the HMDSO bubbler at 0.34 L min^−1^. The particles were collected on a glass fiber filter (Albert LabScience, Germany) with the aid of a Mink MM 1144 BV vacuum pump (Busch, Sweden).

### APTES‐Conjugation (**2**)

FSP‐made SPIONs (10 mg) were dispersed in DMF (10 mL) by ultrasonication using a Vibra‐Cell sonicator with a 13 mm probe tip (Sonics, Newton, CT, USA) operating at 20% amplitude, pulse 10 sec on and 1 sec off, for 3 min. APTES (either 60, 200, or 600 µL) (Sigma–Aldrich, Sweden) was added to the particle suspensions in order to produce SPIONs modified with 0.6 vol%, 2 vol% or 6 vol% APTES, respectively. The reaction was left overnight at 75 °C under rotation. The particles were washed by centrifugation (20 000 g, 10 min, 21 °C), removal of supernatant, and re‐dispersion in fresh MeOH. The washing step was repeated a total of three times. Particles were dried at 60 °C for at least 2 h prior to characterization and further functionalization.

### Quantification of Surface Accessible Amines Using FMOC

SPIONs were modified with 0.6 vol%, 2 vol%, or 6 vol% of APTES as described above. APTES‐modified SPIONs (0.6 vol% APTES, 60 µL, 0.26 mmol, 1 equiv.) were dispersed in DMF to achieve a final concentration of 1 mg mL^−1^ by repetitive rounds of ultrasonication using a water bath (Elmasonic, USA) and cup horn ultrasonicator operating at 90% amplitude for 1 min until no aggregates were observed. *N,N*‐Diisopropylethylamine (DIPEA) (178 µL, 1,0 mmol, 4 equiv.) was added to the suspension, followed by Fmoc‐Cl (132 mg, 0.51 mmol, 2 equiv.). The reaction was left overnight at room temperature under rotation. The same procedure was followed to prepare Fmoc‐protected APTES‐modified SPIONs of 2 vol% and 6 vol% APTES, details are available in (Table S1, Supporting Information).

The particles were washed by centrifugation (20 000 g, 10 min, 21 °C), removal of supernatant and re‐dispersion in fresh MeOH. The washing step was repeated a total of three times. Particles were dried at 60 °C for at least 2 h prior to Fmoc removal. The Fmoc‐deprotection was carried out by suspending the particles in piperidine (20% v/v) in DMF using repetitive rounds of ultrasonication using a water bath and cup horn ultrasonicator operating at 90% amplitude for 1 min until no aggregates were observed. The reaction was left under rotation at room temperature for 2 h. Particles were centrifuged and the supernatant was collected for analysis.

The calibration curve was prepared using Fmoc‐Val‐OH stock solutions (500 µg mL^−1^) mixed with piperidine (20% v/v) in DMF and diluting to appropriate concentrations. Fmoc removal reactions were stirred for 30 min at room temperature. All samples were quantified using a UV–vis spectrophotometer (Agilent, Santa Clara, CA USA) at 301 nm (n = 3).

### Synthesis of 6‐heptynoic Acid Succinimidyl Ester (**4**)

6‐hepynoic acid (200 mg, 1.58 mmol, 1 equiv) (Sigma–Aldrich, Sweden) was dissolved in anhydrous dichloromethane (20 mL). *N*‐hydroxysuccinimide was added to the reaction flask (456 mg, 4.05 mmol, 2.5 equiv) followed by 1‐(3‐dimethylaminopropyl)‐3‐ethylcarbodiimide hydrochloride (EDC, 454 mg, 2.37 mmol, 1.5 equiv) (Fisher Scientific, Sweden). The reaction was stirred overnight at room temperature. Saturated NaHCO_3_ was added to the reaction flask, and the aqueous layer was extracted with ether. The combined organic layer was washed with water and brine (twice) and then dried over Na_2_SO_4_. The solution was concentrated under reduced pressure to afford the product as a white/yellow solid (331 mg, 1.48 mmol 94% yield). The product was used in the next step without further purification.

^1^H NMR (400 MHz, Chloroform‐*d*) δ 2.84 (s, 4H), 2.65 (t, *J* = 7.4 Hz, 2H), 2.25 (td, *J* = 6.9, 2.7 Hz, 2H), 1.97 (t, *J* = 2.7 Hz, 1H), 1.93 – 1.84 (m, 2H), 1.70 – 1.61 (m, 2H).

### Conjugation of 6‐heptynoic Acid Succinimidyl Ester (**4**) and alkyne‐PEG5‐N‐hydroxysuccinimidyl Ester (**9**) onto SPIONs (**5** and **10**)

APTES‐modified SPIONs (10 mg) were dispersed in DMSO (7 mL) using repetitive rounds of ultrasonication using a water bath and cup horn ultrasonicator operating at 90% amplitude for 1 min until no aggregates were observed. Triethylamine (100 µL) was added to the suspension. 6‐heptynoic acid succinimidyl ester (30 mg, 0.13 mmol) or alkyne‐PEG5‐*N*‐hydroxysuccinimidyl ester (30 mg, 0.07 mmol) (Sigma–Aldrich, Sweden) were dissolved in DMSO (3 mL) and then added to the particle suspension. The mixtures were ultrasonicated using a Vibra‐Cell sonicator with a 13 mm probe tip operating at 20% amplitude, pulse 10 sec on and 1 sec off, for 3 min. The reactions were left overnight at 50 °C under rotation. The particles were washed by centrifugation (20 000 g, 20 min, 21 °C), removal of supernatant and re‐dispersion in fresh MeOH. The washing step was repeated a total of three times. Particles were dried at 60 °C for at least 2 h prior to characterization and further functionalization.

### Quantification of Alkynes on SPION Surface

Alkyne‐modified SPIONs (5 mg) were dispersed in DMSO (5 mL) using repetitive rounds of ultrasonication using a water bath and cup horn ultrasonicator operating at 90% amplitude for 1 min until no aggregates were observed. Sodium ascorbate (5 mg) (Sigma–Aldrich, Sweden) and CuSO_4_ (5 mg) (Sigma–Aldrich, Sweden) were added to each suspension, followed by azide‐containing fluorophore (167 µL, 1 mg mL^−1^ in DMSO) (Invitrogen, CA, USA) with emission/excitation wavelengths at 519/495 nm. The reactions were left in the dark overnight at room temperature under rotation. The particles were washed by centrifugation (20 000 g, 20 min, 21 °C), removal of supernatant and re‐dispersion in fresh MeOH. The washing step was repeated a total of three times. Particles were dried at 60 °C for at least 2 h prior to analysis of fluorescent intensity using Spark plate (Tecan, Austria). A calibration curve was prepared using known concentrations of the fluorophore as standard samples.

### Azide‐Modification of Antibodies (**7**)

Mouse monoclonal ICAM1 antibodies (ab171123, Abcam, Cambridge, UK) were modified with an azide following the experimental protocol as provided by the manufacturer using Invitrogen SiteClick Antibody Azido Modification kit (Invitrogen, CA, USA).

### Antibody‐Conjugation (**8** and **11**)

Alkyne‐modified SPIONs (5 mg) were dispersed in 1xTris buffer (5 mL) using a cup horn ultrasonicator operating at 90% amplitude for 1 min. The sonication was repeated until no aggregates were observed. Sodium ascorbate (5 mg) (Sigma–Aldrich, Sweden) and CuSO_4_ (5 mg) (Sigma–Aldrich, Sweden) were added to the suspensions. Azide‐modified antibodies (100, 200, or 500 ug) were added to the suspensions and the reactions were left overnight at room temperature under rotation. Particles were washed using magnetic decantation and resuspension in fresh PBS a total of three times.

### X‐Ray Diffraction (XRD)

The crystalline structure of the pristine nanoparticles, SiO_2_‐coated γ‐Fe_2_O_3_, was studied using an X‐ray diffractometer (D2 Phaser, Bruker, Germany) with a LYNXEYE XE‐T detector. XRD patterns were obtained using Cu‐K_α_ radiation (0.154 nm) at 30.0 kV and 10.0 mA. The pattern was measured between 20.0° and 80.5° 2θ at a step size 0.01° 2θ. The background was subtracted and the baseline smoothened using DIFFRAC.EVA software.

### Attenuated Total Reflectance Fourier‐Transform Infrared (ATR‐FTIR) Spectroscopy

To study the surface functional groups after each modification step, nanoparticles were analyzed using an ATR‐FTIR spectrometer (Alpha II, Bruker, Germany) equipped with a diamond crystal. The particles were dried at 60 °C for at least 2 h prior to analysis. The measurements were performed at a resolution of 4 cm^−1^, within the range of 4000–400 cm^−1^, at room temperature. The baseline was corrected for each spectrum using the baseline correction function of OPUS software.

### Transmission Electron Microscopy (TEM)

The nanoparticles were dispersed in EtOH at a concentration of 0.01 mg mL^−1^ and sonicated for 5 min at 90% amplitude using a cup horn ultrasonicator (Sonics), vortexing for 10 s every 1 min. A 5 µL droplet of the nanoparticle dispersion was placed on a Formvar/Carbon 300 square mesh copper grid (Delta Microscopies, France). A FEI Titan Themis 200 equipment (Thermo Fisher Scientific, USA) operating at 200 kV was then used to visualize the particles through TEM. Once the micrographs were obtained, ImageJ software was used to measure the diameter of 100 particles. The Sturges method was used to determine bin number and plot a histogram of the distribution.^[^ ^88^ ^]^ The sample was determined to be log‐normally distributed according to the Shapiro–Wilk test for normality, therefore, the geometric mean and geometric standard deviation from the fitting of the log‐normal curve were used to determine the particle size.

### Thermogravimetric Analysis (TGA)

The amount of organic material on the SPION surface for different functionalization steps was characterized by a thermogravimetric analyzer (Discovery, TA Instruments Ind., USA). The experiments were conducted under a nitrogen gas purge of 20 mL min^−1^ and a heating rate of 10 °C min^−1^ from ambient temperature to 900 °C. All data was normalized to 120 °C. Each experiment was conducted in at least triplicates (n ≥ 3).

### Dynamic Light Scattering (DLS)

The hydrodynamic diameter was measured by dynamic light scattering (Litesizer 500, Anton Paar, Austria). The suspensions were diluted to achieve a concentration of 1 mg mL^−1^ in either DMF, DMSO, or water (Table 1) prior to measurements. Each measurement was conducted in triplicates (n = 3), except for the antibody‐conjugated particles, which were conducted in one single measurement due to the rapid sedimentation of particles. A quartz cuvette was used for all measurements.

### Gel Electrophoresis (SDS‐PAGE)

Particles were bioconjugated with ICAM1‐targeting antibodies, both in the presence and absence of a copper catalyst. Bioconjugated particles dispersed in PBS (1 mg mL^−1^) were diluted in a 1:2 volume ratio in a non‐reducing SDS buffer. This buffer consisted of 0.5 m Tris‐HCl (pH 6.8), 25% v/v glycerol, 2% (w/v) SDS, and 0.1% (w/v) bromophenol blue. The samples were then heated at 95 °C for 5 min. A standard curve was made to quantify the adsorbed proteins on the particles. Relevant concentrations of the azide‐modified antibody were diluted in a 1:2 volume ratio of the antibody and the non‐reducing SDS buffer to obtain a final loading of 1, 0.5, 0.25, and 0.1 µg of antibody on the gels. Volumes of 10 µL of the standard curve and the samples were pipetted onto wells of Any kD Mini‐Protean TGX Stain‐Free 15W Precasted gels (Bio‐Rad Laboratories AB, CA, USA) at least in triplicates. Furthermore, 4 µL Precision Plus Protein Unstained Protein Standards (Bio‐Rad Laboratories AB, CA, USA) were used as molecular weight markers in every gel. The gel electrophoresis was run at 200 V for 30 min at 4 °C in a Mini‐Protean Tetra cell (Bio‐Rad Laboratories AB, CA, USA) using Tris/glycine/SDS running buffer (Bio‐Rad Laboratories AB, CA, USA). After the run, the gels were rinsed with distilled water and analyzed using the GelDoc Go Gel imaging system (Bio‐Rad Laboratories AB, CA, USA) with a 5 min activation and 3 s exposure. Finally, the Image Lab Software (Bio‐Rad Laboratories AB, CA, USA) was used to analyze the gel images. The intensity profiles of each gel lane were obtained, and densitometry analysis with the peak area was done to quantify the adsorbed antibodies.^[^ ^89^ ^]^

### Cell Maintenance and In Vitro Inflammation Model

Caco‐2 cells (originally obtained from the American Type Culture Collection), passage 95–105, were maintained in Dulbecco's modified Eagle's medium, containing 10% (v/v) fetal bovine serum, and 1% (v/v) nonessential amino acids. The cells were cultured in an incubator at 37 °C, 10% CO_2_ (MMM Group, Munich, Germany) while maintained in 75 cm^2^ tissue culture flasks. Cells were seeded on the bottom side of Transwell polycarbonate filters (Corning, NY, USA; diameter 6.5 mm, pore size 0.4 µm) at a density of 1.5 × 10^5^ cells/filter and maintained in Dulbecco's modified Eagle's medium with 10% fetal bovine serum, 1% nonessential amino acids, 100 U mL^−1^ penicillin, and 100 µg mL^−1^ streptomycin prior to the experiment. All cell culture media and reagents were purchased from ThermoFisher Scientific (Waltham, MA, USA) or Sigma–Aldrich (St. Louis, MO, USA). After 21 days in culture, the cells were treated with a mixture of inflammatory mediators by application on the basolateral side of the cells. The mixture included tumor necrosis factor (TNF)‐α (50 ng mL^−1^), interleukin (IL)‐1β (25 ng mL^−1^), lipopolysaccharides (LPS; 10 ng mL^−1^), and interferon ((IFN)‐γ (50 ng mL^−1^) in culture medium.^[^ ^80^ ^]^ After 24 h, filters were apically exposed to suspensions of SPIONs. The particles were suspended in Hank's Balanced Salt solution (HBSS) at a concentration of 500 µg mL^−1^ and re‐dispersed by shaking, prior to exposure to cells. The cells were then exposed to the particles for 2 h under shaking (500 rpm) at 37 °C. The filters were then washed with HBSS for 5 min under shaking (300 rpm) at 37 °C. The washing step was repeated a total of three times. TEER was recorded in HBSS for all filters.

### Cytotoxicity

Caco‐2 cells were plated into black opaque 96‐well plates at a density of 5 × 10^4^ cells per well in 300 µL of culture medium. The cells were allowed to attach for 24 h before treatment. Subsequently, the culture medium was replaced by 100 µL of particle suspensions in six replicates per treatment and incubated for 24 h (at 37 °C, 10% CO_2_). Positive controls were prepared by diluting 10% (w/v) sodium dodecyl sulfate (SDS) in water to achieve a final concentration of 0.22% (v/v) SDS in the cell culture medium. The culture medium was used as a negative control. The water content in all treatments was kept at or below 2%. Finally, the viability of Caco‐2 cells was evaluated with the CellTiter‐Glo luminescent cell viability assay (Promega, USA). The luminescent signal from each well was determined with a plate reader (Tecan, Austria). Microsoft Excel software was used for the analysis of significance.

### Quantification of Fe in SPION‐Exposed Cells with ICP‐OES

Following SPION exposure and washing, the filters were excised and dissolved in 1m NaOH at 60 °C overnight under rotation. The mixtures were centrifuged (20 000 g, 10 min, 21 °C) and the supernatant was discarded, followed by resuspension in 300 µL of 12m HCl. Each sample was heated to 80 °C for 1 h. The samples were diluted 10–15 times with milli‐Q water containing 5% HNO_3_ and filtered with 0.2 µm syringe filters (Whatman) before measurement. Avio 200 Scott/Cross‐Flow Configuration was used for ICP measurements. A calibration curve was performed for the measurements using a Multielement Calibration Standard (CPAchem). Concentrations of 0, 0.1, 0.25, and 0.5 ppm of Fe were used to create a 4‐point linear regression. All measured values were within a *relative standard deviation* (RSD) of 0.2–3.6%. In total, samples of filter‐grown cells exposed to HBSS (control), SPION‐linker, SPION‐linker‐Ab, SPION‐PEG, and SPION‐PEG‐Ab were prepared and analyzed (n ≥ 4 for each group). Microsoft Excel software was used for the analysis of significance.

### Staining of Inflamed Cells and Confocal Laser Microscopy Imaging

Caco‐2 cells were grown on the bottom side of Transwell filters, treated with inflammatory agents, exposed to SPIONs, and washed as described above. Filters were fixed in 4% paraformaldehyde for 15 min at room temperature and permeabilized with 0.2% Triton‐X‐100 for 15 min at room temperature. Filters were rinsed with PBS three times and then incubated in a 3% BSA solution in PBS with 0,1% Tween 20 (PBST) (Sigma–Aldrich) for 1 h at room temperature to block non‐specific binding. Every filter was incubated with rabbit recombinant monoclonal anti‐ICAM1 antibody (ab282575, Abcam, Cambridge, UK) (diluted 1:500 in 1% BSA in PBST) for 1 h in darkness at room temperature. Filters were washed with PBST for 5 min, three times. Filters were then incubated with Alexa Fluor 555 donkey anti‐rabbit (Invitrogen, CA, USA) (diluted 1:500 in 1% BSA in PBST) for 1 h in darkness at room temperature. Filters were washed with PBST for 5 min, three times and then nuclei were stained with Hoechst (diluted 1:10 000) for 15 min in darkness at room temperature. Filters were washed with PBS for 5 min, three times. Images were acquired using laser scanning confocal microscopy (Zeiss LSM 780 with a 63× objective; Zeiss ZEN software; Zeiss, Oberkochen, Germany).

## Conflict of Interest

The authors declare no conflict of interest.

## Supporting information



Supporting Information

## Data Availability

The data that support the findings of this study are available from the corresponding author upon reasonable request.
