# Supplementary material for: Click Chemistry‐Based Bioconjugation of Iron Oxide Nanoparticles
Source: Small. 2025 Feb 9;21(11):2407883. doi: 10.1002/smll.202407883 (PMC11922026; doi:10.1002/smll.202407883)
Supplement: Supplementary file 1 — Supporting Information [file SMLL-21-2407883-s001.docx]

Supporting Information

Click chemistry-based bioconjugation of iron oxide nanoparticles

Shno Asad, David Ahl, Yael del Carmen Suárez-López, Máté Erdélyi, Mia Phillipson, Alexandra Teleki*


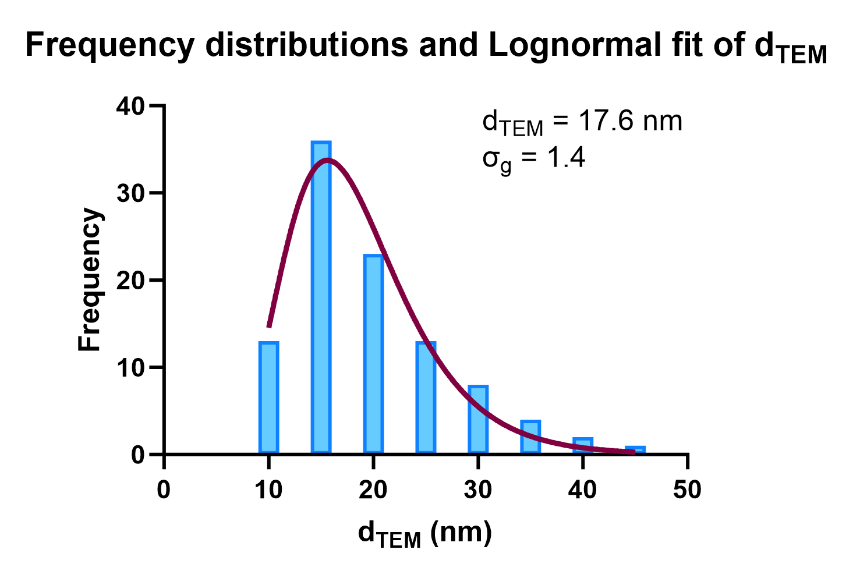


**Figure S1.** Primary particle size distribution of SiO_2_-coated γ-Fe_2_O_3_ nanoparticles determined by particle counting in TEM images. The solid line represents the log-normal size distribution fit.


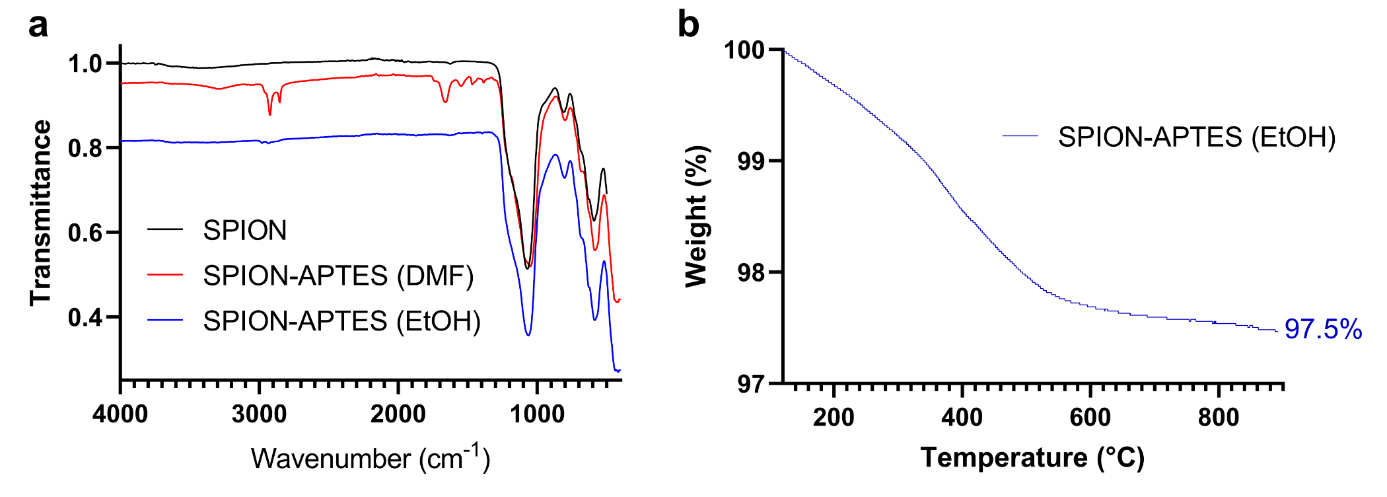
**Figure S2.** APTES-conjugation in EtOH. (a) ATR-FTIR spectra of APTES-reaction performed in EtOH (blue) showing none of the characteristic APTES functional groups. (b) TGA profile demonstrated no additional weight reduction compared to non-modified SPIONs.


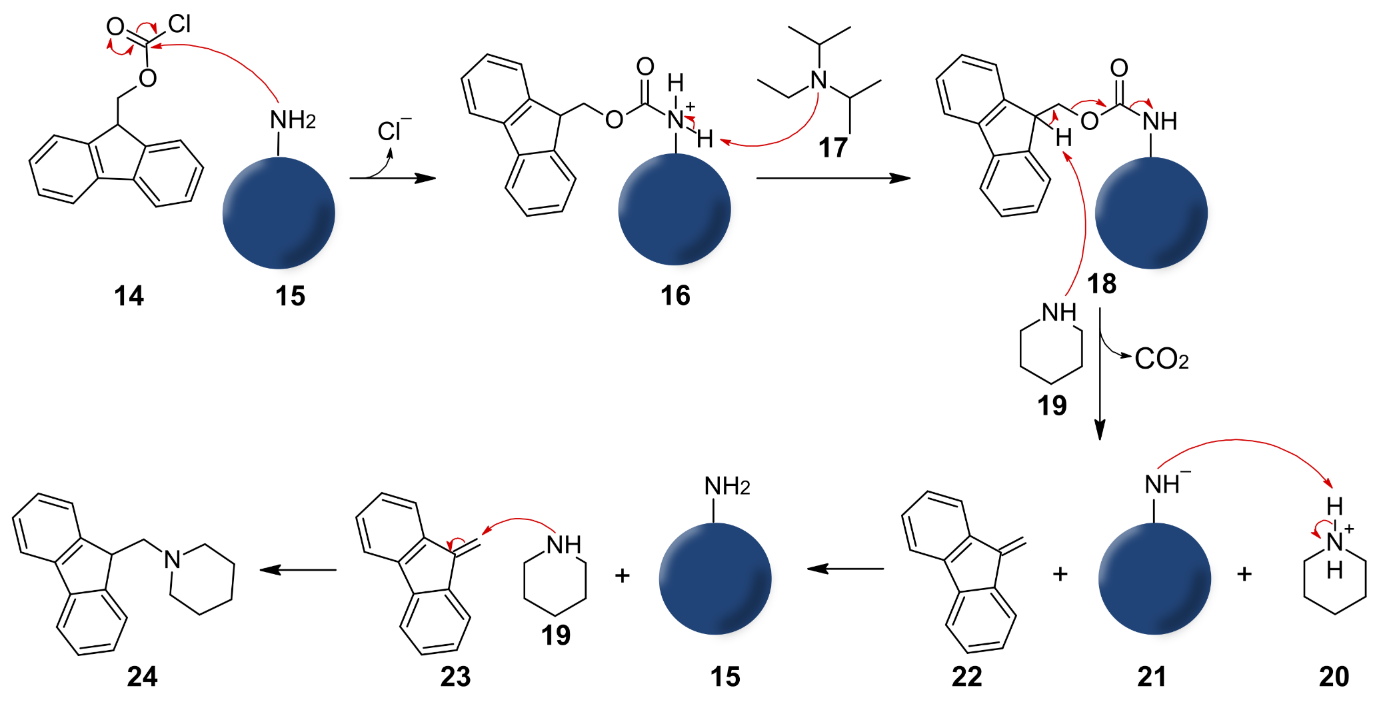


**Figure S3.** Reaction mechanism of Fmoc-protection and de-protection of surface accessible NH_2_-groups on the APTES-modified SPIONs.


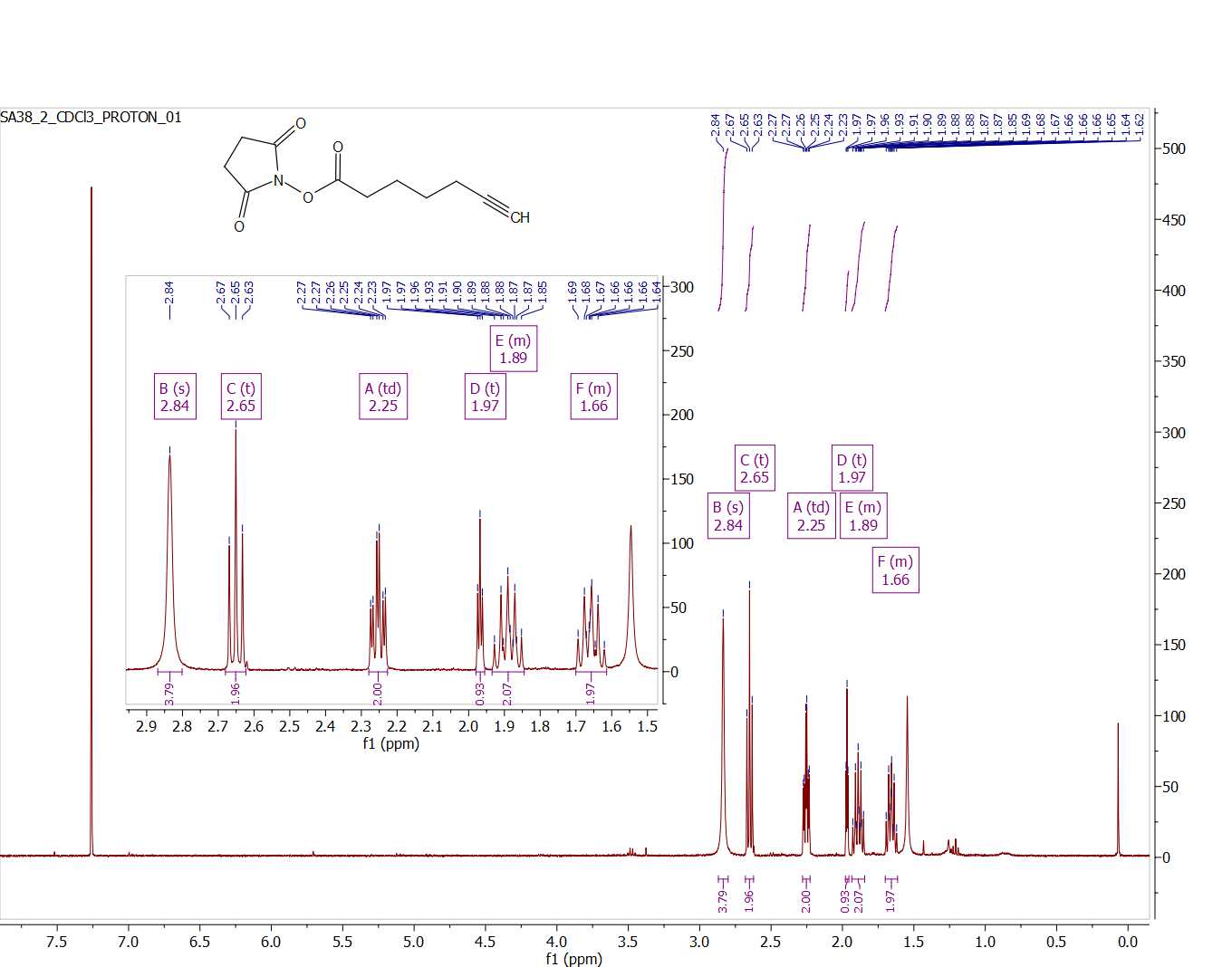


**Figure S4.** H^1^ NMR spectrum of an organic linker containing a terminal alkyne.


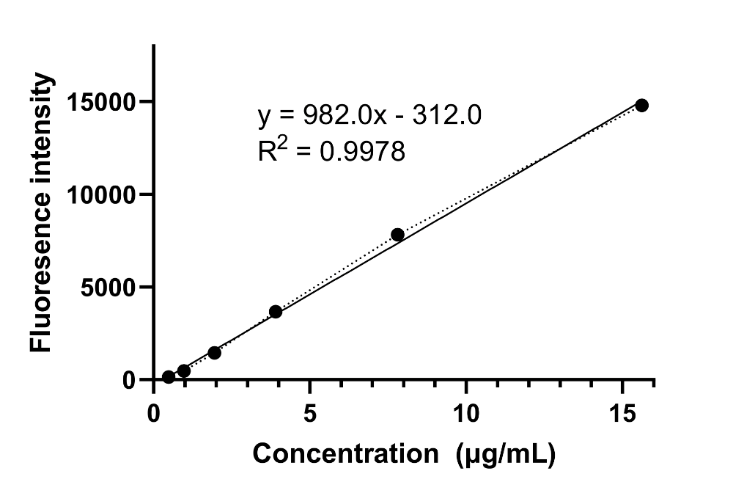


**Figure S5.** Standard curve of fluorescent intensity of Alexa Fluor™ 488 Azide, used for quantification of linker and PEG on SPIONs.

**
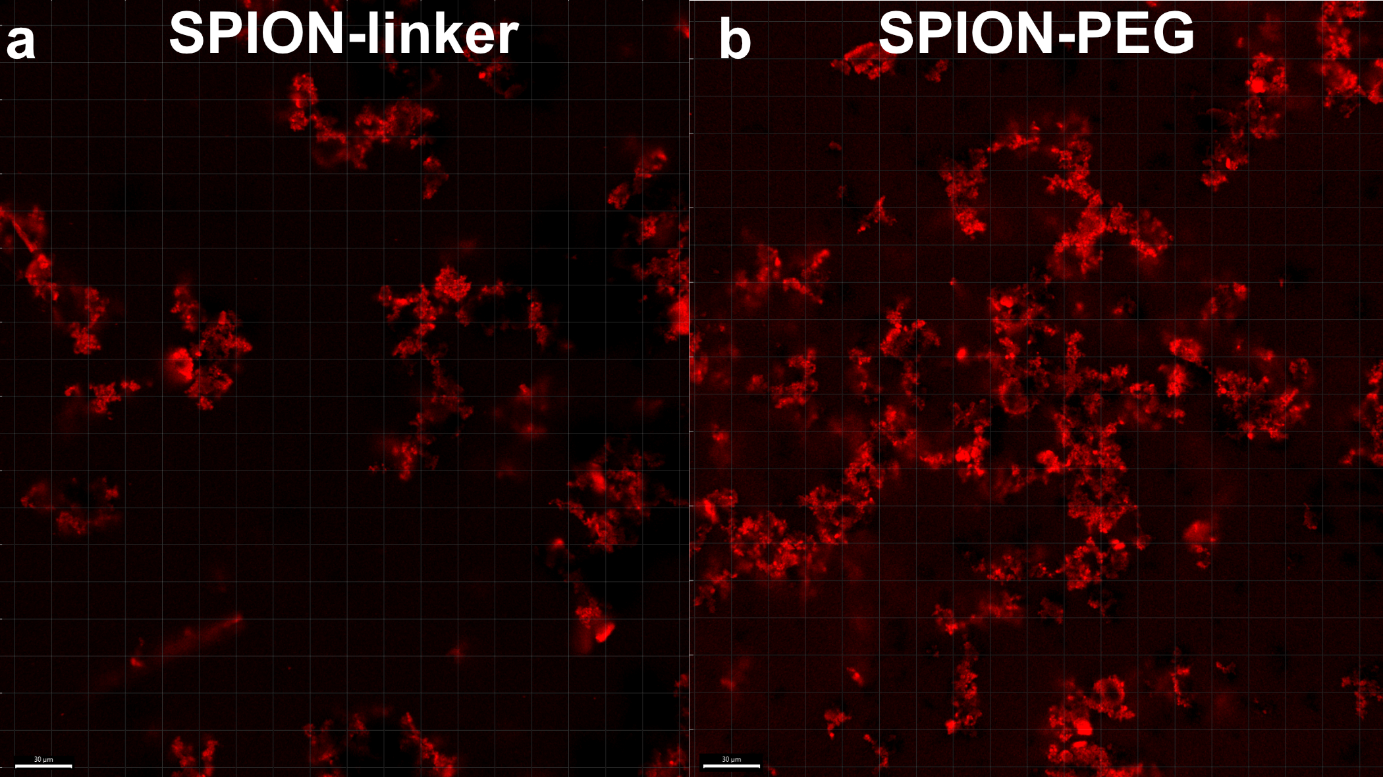
**

**Figure S6.** SPIONs labelled with fluorophore *via* copper catalyzed click chemistry. Linker-conjugated (a) and PEG-conjugated (b) SPIONs were tagged with an azide-containing fluorophore (Alexa Fluor™ 488 Azide) to confirm presence of the alkyne moiety (scale bar: 30 µm).


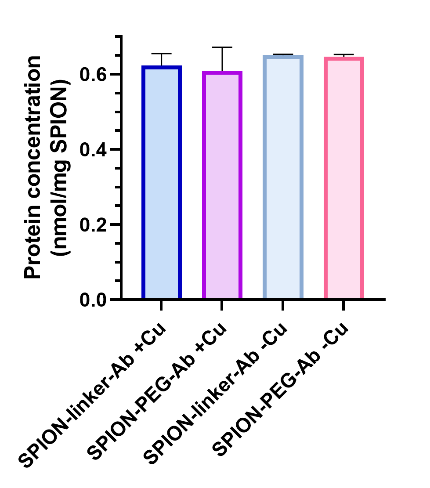


**Figure S7.** BCA assay quantification of antibodies on SPIONs modified with 100 µg/mL antibodies (n ≥ 2).


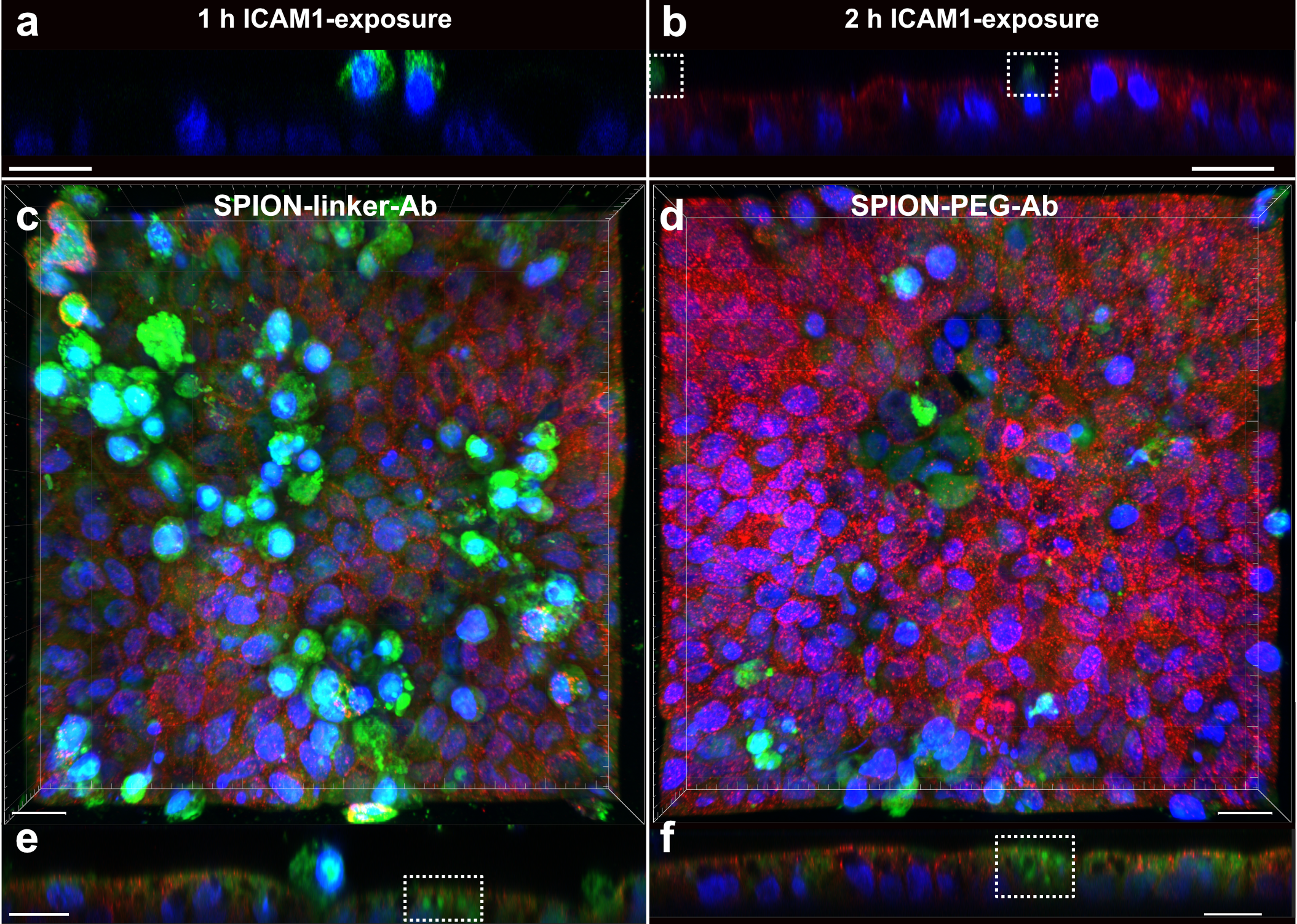


**Figure S8**. Inflamed cells Caco-2 cell monolayer cultured in Transwell filters and stained for ICAM1 (red) and nuclei (blue). Cells exposed to free, fluorescently-tagged anti-ICAM1 antibodies (green) for 1 h (a) and 2h (b). Overview (c, d) and cross-sectional (e, f) images of inflamed cells exposed to bioconjugated SPIONs (green), prepared with 20 µg/mL of added antibodies. (scale bar: 20 µm). Particle aggregates (e, f) and vague green signal from antibodies (b) are indicated in squares.

**Table S1**. Amount of APTES, DIPEA and Fmoc-Cl used in reactions to prepare 0.6 vol%, 2 vol% and 6 vol% APTES-modified SPIONs.

|  | APTES | DIPEA | Fmoc-Cl |
| --- | --- | --- | --- |
| 0.6 vol% APTES | 60 µL, 0.26 mmol,  1 equiv. | 178 µL, 1,0 mmol,  4 equiv. | 132 mg, 0.51 mmol,  2 equiv. |
| 2 vol% APTES | 200 µL, 0.85 mmol,  1 equiv. | 466 µL, 3,4 mmol,  4 equiv. | 441 mg, 1.7 mmol,  2 equiv. |
| 6 vol% APTES | 600 µL, 2.6 mmol,  1 equiv. | 1.78 mL, 10.2 mmol, 4 equiv. | 1.3 g, 5.13 mmol,  2 equiv. |

**Table S2**. SDS-PAGE data of adsorbed antibodies (µg/mg SPION). All reactions were performed in three replicates, either in presence or absence of copper catalyst.

|  | **+ Cu** | | | **- Cu** | | |
| --- | --- | --- | --- | --- | --- | --- |
|  | *Rep. 1* | *Rep. 2* | *Rep. 3* | *Rep. 1* | *Rep. 2* | *Rep. 3* |
| **Linker-Ab 20 µg/mL** | 2.12 | 0.81 | 0.59 | 3.65 | 1.84 | 1.23 |
| **Linker-Ab 40 µg/mL** | 8.62 | 0.37 | 0.80 | 25.19 | 2.23 | 3.17 |
| **Linker-Ab 100 µg/mL** | 24.35 | 7.01 | 13.78 | 69.45 | 18.16 | 19.87 |
| **PEG-Ab 20 µg/mL** | 1.37 | 0.82 | 0.62 | 10.01 | 16.74 | 1.44 |
| **PEG-Ab 40 µg/mL** | 2.17 | 2.77 | 2.20 | 20.55 | 26.50 | 5.22 |
| **PEG-Ab 100 µg/mL** | 24.04 | 15.10 | 12.78 | 60.94 | 37.89 | 36.43 |

**Table S3.** TEER values across cell monolayers of healthy and inflamed Caco-2 cells. Values are represented as means ± SD.

| **Application** | **Model** | **TEER (Ω cm^2^)** | **Number of filters** |
| --- | --- | --- | --- |
| ICP-OES | Healthy | 266 (± 12.7) | 18 |
|  | Inflamed | 173 (± 8.1) | 18 |
| Microscopy | Healthy | 305 (± 9.6) | 6 |
|  | Inflamed | 190 (± 3.8) | 6 |

**Calculation for estimating antibodies on nanoparticle surface**

Below is an example of the calculation used to estimate the number of antibodies on SPION-PEG-Ab, based on TGA data. The numbers highlighted in **bold** are constants also used to calculate antibody numbers for other bioconjugated particles prepared in this work.

- γ-Fe₂O₃ has a density of ~**5.24 g/cm³**
- SiO₂ has a density of ~**2.65 g/cm³**
- Density of 23 wt% SiO_2_-coated γ-Fe_2_O_3_ nanoparticle (ρ_SiO2@Fe2O3_):

$$\frac{1}{\rho_{{SiO}_{2}@{Fe}_{2}O_{3}}}= \frac{wt fraction {Fe}_{2}O_{3}}{\rho_{{Fe}_{2}O_{3}}}+ \frac{wt fraction {SiO}_{2}}{\rho_{{SiO}_{2}}}= \frac{0.77}{5.24}+ \frac{0.23}{2.65}= \boldsymbol{4}\boldsymbol{.}\boldsymbol{278} \boldsymbol{g}/{\boldsymbol{cm}^{\boldsymbol{3}}}$$

- TEM size: **17.56 nm**
- Radius (r): **8.78 nm**
- Nanoparticle volume (V):

$$V=\frac{4\pi r^{3}}{3}= \frac{4*\pi{*8.78}^{3}}{3}=2835 {nm}^{3}=\boldsymbol{2.835*}\boldsymbol{10}^{\boldsymbol{-18}} \boldsymbol{cm}^{\boldsymbol{3}}$$

- Mass of 1 nanoparticle (m_SiO2@Fe2O3_):

$$m_{{SiO}_{2}@{Fe}_{2}O_{3}}=\rho_{{SiO}_{2}@{Fe}_{2}O_{3}}*V= 4.278 g/{{cm}^{3}}* 2.835*{10}^{-18} {cm}^{3}=\boldsymbol{1}\boldsymbol{.}\boldsymbol{21}\boldsymbol{*}\boldsymbol{10}^{\boldsymbol{-}\boldsymbol{17}} \boldsymbol{g}$$

- TGA weight loss SPION-PEG-Ab (+Cu): 5.4 wt%
- Mass antibody (m_Ab_) per nanoparticle:

$$m_{Ab}= m_{{SiO}_{2}@{Fe}_{2}O_{3}}*wt fraction= 1.21*{10}^{-17} g *0.054=6.55*{10}^{-19}g$$

- Molecular weight (MW_Ab_) of IgG antibody: **150 000 g/mol**
- Moles of antibody (mol_Ab_) per nanoparticle:

$${mol}_{Ab}= \frac{m_{Ab}}{{MW}_{Ab}}= \frac{6.55*{10}^{-19} g}{150 000 g/mol}=4.37*{10}^{-24} mol$$

- Number of antibodies per nanoparticle:

$${mol}_{Ab}*Avogadro constant= 4.37*{10}^{-24} mol*6.022*{10}^{23}=2.63 antibodies$$

Below is an example of calculations used to estimate the number of adsorbed antibodies on SPION-PEG-Ab, based on SDS-PAGE data. The number highlighted in bold are constants also used to calculate adsorbed antibodies numbers for other bioconjugated nanoparticles prepared in this work.

- Number of nanoparticles in 1 mg of SPIONs (N_NP_):

$$N_{NP} = \frac{1 mg}{m_{{SiO}_{2}@{Fe}_{2}O_{3}}}= \frac{0.001 g}{1.21*{10}^{-17} g}= \boldsymbol{8}\boldsymbol{,}\boldsymbol{25}\boldsymbol{*}\boldsymbol{10}^{\boldsymbol{13}}$$

- SDS-PAGE quantification of adsorbed antibodies on SPION-PEG-Ab (+Cu): 17.3 µg/mg SPION.
- Mass adsorbed antibodies per nanoparticle (Ads_Ab_):

$${Ads}_{Ab}= \frac{17.3*{10}^{-6} g}{8,25*{10}^{13}}=2.1*{10}^{-19} g$$

- Molecular weight (MW_Ab_) of IgG antibody: **150 000 g/mol**
- Moles of antibody (mol_Ab_) per nanoparticle:

$${mol}_{Ab}= \frac{m_{Ab}}{{MW}_{Ab}}= \frac{2.1*{10}^{-19} g}{150 000 g/mol}=1.4*{10}^{-24} mol$$

- Number of antibodies per nanoparticle:

$${mol}_{Ab}*Avogadro constant= 1.4*{10}^{-24} mol*6.022*{10}^{23}=0.84 antibodies$$
